# A qualitative investigation of social service workers’ experiences with compassion training in the workplace

**DOI:** 10.3389/fpsyg.2025.1593026

**Published:** 2026-02-02

**Authors:** Anneli O. Borgen, Eline L. Håkedal, Nanja H. Hansen

**Affiliations:** 1 Psykiatrien Vest Region Sjælland, Holbæk, Denmark; 2 Socialcenter Lillebælt i Region Syd Danmark, Fredericia, Denmark; 3 Department of Clinical Medicine, Aarhus University, Danish Center for Mindfulness, Aarhus, Denmark

**Keywords:** acceptability, compassion training, mindfulness, challenges, implementation, social care work

## Abstract

**Objectives:**

This study aimed to qualitatively explore the experiences of professionals in social services who completed a compassion training intervention in their workplace. The focus was on understanding how contextual factors influence the implementation of such an intervention. Additionally, the study sought to investigate how the professionals perceive and apply compassion and compassion-related skills after completing the training, with the aim of gaining insights into how they incorporate the competencies from the program into their daily lives both at the micro (personal experience) and macro (implementational barriers) level.

**Methods:**

Seven participants (four pedagogical staff workers and three social workers) from a municipal institution in Denmark, who had completed the compassion training in their workplace, were recruited and semi-structured interviews conducted. The data was analyzed using Thematic Analysis.

**Results:**

The analysis identified four themes relating to the acceptability of compassion training including: (1) Challenges related to meditation; (2) Follow-up and maintenance; (3) Vulnerability, feelings of psychological safety, and inter-connectedness; (4) and Task overload as a challenge to the implementation of compassion training. The professionals’ understanding and use of (self)compassion were grouped into three themes: (1) Boundaries, (2) Mindfulness, and (3) Self-care as a form of self-compassion.

**Conclusion:**

The study emphasizes the importance of a supportive environment, managing task overload, and organizational commitment to the implementation of compassion training programs aimed at social care workers.

## Introduction

Professionals in the social and health care sector are at heightened risk for stress, burnout, and compassion fatigue, due to the high emotional demands of their work (Nagle et al., 2024; Garnett et al., 2023). Compassion fatigue, a term coined by Figley (1995), has been defined as the reduced capacity to be empathetic or emotionally involved in suffering because of prolonged exposure to traumatized individuals. This concept is closely linked to the term empathic distress, which results from a perceived lack of resources to cope with the emotional experiences of others and oneself (Klimecki et al., 2014; Hofmeyer et al., 2020). The concept is also related to burnout, which involves emotional exhaustion and depersonalization, often resulting from chronic stress (Nagle et al., 2024; Hofmeyer et al., 2020). These challenges have prompted initiatives for preventative measures, such as interventions focusing on facilitating mindfulness and compassion. Mindfulness involves being aware of the present moment and maintaining a non-judgmental, attentive attitude towards it (Kabat-Zinn, 1994), while compassion may be defined as a recognition of suffering coupled with a desire to alleviate it, resulting in prosocial behaviors (Gilbert and Van Gordon, 2023; Gilbert, 2017).

Mindfulness- and compassion-based interventions (MBIs and CBIs) have demonstrated effectiveness in enhancing individual’s well-being and in reducing psychological distress, and stress (Kirby et al., 2017; Hansen et al., 2021; Conversano et al., 2020; van Agteren et al., 2021; Rojas et al., 2023), as well as improving trait mindfulness and self-compassion (Kirby et al., 2017), prosocial behavior (Luberto et al., 2018), and aspects of emotion regulation (Hansen et al., 2021; Rojas et al., 2023; Jazaieri et al., 2018). Furthermore, CBI’s have been found to lead to increased altruistic orientation, such as empathic concern for others (Brito-Pons et al., 2018; Andreu et al., 2025). Compassion training in organizations has shown promising effects of decreasing stress and mental illness, and increasing self-compassion (Andersson et al., 2022). Studies suggest that both compassion satisfaction, which refers to the positive feelings derived from helping others (Conrad and Kellar-Guenther, 2006), and self-compassion, the concept of having compassion for ones own suffering (Neff, 2023), represent protective factors against compassion fatigue and secondary traumatization among care professionals and are associated with well-being and quality of patient care (Conversano et al., 2020; Kinman and Grant, 2020).

While research highlights the mental health benefits of compassion training, studies exploring the inhibitors and facilitators to implementing CBIs in the workplace remain limited (Paakkanen et al., 2024). Preliminary findings have identified factors that could influence their acceptability. The daily meditation assignments, a core element of MBIs and CBIs, are linked to challenges in adherence, as well as maintaining attention throughout the practices (Kropp and Sedlmeier, 2019; Watts et al., 2021). Additionally, the commitment required for an intervention spanning several weeks has been identified as a significant barrier to workplace engagement (Watts et al., 2021; Lamothe et al., 2018). Sinclair et al. (2021) have also noted the importance of maintaining the learning outcomes from the training as important for the continued effect and application of the knowledge taught within CBIs. The group-based format of the interventions has been found to enhance participant learning (Coholic et al., 2019) though the presence of leaders may inhibit open sharing of personal experiences (Farr and Barker, 2017).

Research on the effects of compassion training specifically, within the social service system, is scarce. One qualitative study found that compassion was viewed as a central component of professionals within the field of social work and imperative to ethical practice (Clark et al., 2023). Self-compassion is shown to be a significant predictor of both personal and professional self-care among social workers (Miller et al., 2019). Empirical findings suggest that social workers are moderately self-compassionate, and the degree of their self-compassion is influenced by factors such as years of experience, health status, and economic resources, indicating that professionals with less experience and lower resources are less self-compassionate (Miller et al., 2019). Furthermore, focus on the importance of self-care and self-compassion is largely overlooked in social care education (Iacono, 2017).

Given the limited understanding of perceived inhibitors and facilitators to implementing compassion training in workplace settings, as well as the sparse knowledge about how professionals conceptualize and experience ^1^(self-) compassion after such training, this study aims to address these gaps by qualitatively investigating professionals’ perceptions of the implementation of a compassion training intervention in their workplace, and exploring their understanding of the concept of (self-) compassion and their experiences of integrating compassion practices into their daily lives following participation in the intervention.

## Method

### Data collection

An open, inductive approach was chosen for the present qualitative study, as opposed to a more hypothesis-driven deductive approach. This decision was based on the limited pre- existing knowledge available on these subjects, which deemed it desirable to remain open and free from assumptions when investigating these objectives. Consistent with principles of qualitative methodology, this study aimed to elucidate the perceptions of formal care providers who received compassion training in their workplace. Unlike quantitative approaches, which seek to establish cause-effect relationships and make predictions, qualitative methods are not designed to determine how interventions directly lead to specific outcomes (Willig, 2013). Instead, this investigation sought to yield insights into the subjective experience of undergoing such training within a particular context. Qualitative data can offer detailed descriptions of experienced challenges, benefits, and the spectrum of perceptions associated with undergoing compassion training alongside coworkers and leaders. These qualitative accounts are invaluable for obtaining a comprehensive understanding that goes beyond quantifiable data, which is important for advancing the implementation of compassion interventions among care professionals.

### Intervention

The compassion intervention provided to the employees is called Training in Compassion (Danish; Træning i Compassion (TIC)) (Hansen et al., 2025). TIC is an eight-week manualized, research-based program adapted from the American-developed program Compassion Cultivation Training (CCT) (Jazaieri et al., 2013). TIC was adapted from the CCT program to better fit a Danish context and culture (for an in-depth description of the research and adaptation of the TIC program see (Hansen et al., 2025). The program consists of 2-h weekly sessions, delivered in a group setting, and aim to enhance individual mental health and well-being through the cultivation of mindfulness and compassion for oneself and for others (Hansen et al., 2025). By cultivating compassion, individuals may become better equipped to engage with life’s difficulties, or what is often referred to in the program as “suffering.” In TIC, the cultivation of loving-kindness and compassion for one’s own and others’ suffering, is achieved through a combination of meditation practices, dyad and group dialogue regarding own experiences, and psychoeducation concerning mindfulness, body awareness, empathic distress, emotion regulation, self-compassion, and compassion for others (Hansen et al., 2025).

### Participants and recruitment procedure of a larger feasibility study

Participants in this study included social workers and pedagogical staff employed in a Danish municipal social service institution. The pedagogical staff group work flexible hours out in the field, in direct contact and cooperation with the service user and their families, functioning as case managers. The social workers handle formal processing of cases, which include amongst other things implementation and follow up of support measures. The two groups, within the municipal social service institution had been selected by leadership within the institution.

The reasons for selecting these two groups of employees included (1) higher level of stress due to the severity of the cases they were working on, (2) higher risk of burnout and for the social worker group specifically (3) higher risk of prolonged sick-leave and (4) turn-over rate. For these reasons the two groups were selected to get a better understanding of the potential benefits of a compassion training intervention on their well-being. All potential employees had been invited to attend an informational session, where the rationale and details of the study, the intervention, and data management was explained.

Employees of these two groups, including their leaders were subsequently invited to participate in an 8-week compassion training intervention that took place at their place of employment and was conducted within their normal working hours. Potential employees were informed that participation in the training was not mandatory, and any employee could decline the offer with no fear of retribution from leadership.

**Figure 1:**
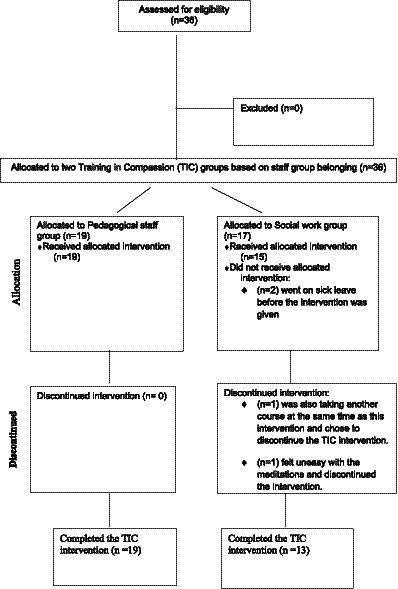
Flowchart of the recruitment for pedagogical and social work staff group. This work is licensed under CC BY 4.0. To view a copy of this license, visit https://creativecommons.org/licenses/by/4.0/

Inclusion criteria included: (1) being an employee (or leadership) within the pedagogical staff group or the social work group, (2) working within this municipal social service institution, (3) all genders, (4) all ages, and (5) all ethnicities. Exclusion criteria included anyone not working within either the pedagogical staff group or the social work group. Of the employees in the pedagogical staff group 19 (*n* = 19) employees decided to participate in the eight-week training and within the social worker group 17 (*n* = 17) employees decided to participate. Figure 1 provides a detailed flowchart of participant enrollment and drop-out. They were provided with oral and written information regarding the study with all information provided at the informational session included in the written information given. They were told that they could take their time to read the informed consent form and if they agreed to participate, they could withdraw their consent at any time. Once they decided to participate, they signed the informed consent form.

### Participants and recruitment for qualitative data and analysis

The qualitative data collected and described here, came from a larger study that aimed to explore the feasibility of providing a compassion-based intervention within a municipal social service institution in Denmark collecting pre-post measures on participants mental health. Inclusion criteria included everyone who had participated in the TIC intervention or had dropped out after the intervention had begun. The only reason for being excluded was if the individual had not participated in the TIC intervention at all. Convenience sampling for the interviews was used and came from the two employee groups: a group of social workers (*n* = 15) and a group of pedagogical staff (*n* = 19). The two groups were similar (see Table 1), yet differences did exist. Differences between the interview groups included that no one in the pedagogical staff group was in the age range of 20–29, while 14.3% was in this age range in the social work group. More men worked as pedagogical staff compared to social work and the social work group had participant drop-out.

**Table 1:** Participants age range, gender, length of education, and ethnicity.

Employee group	Age range	Gender	Education level	Ethnicity other than Danish
Pedagogical staff employee (*n* = 19)	20–29 = 0.0%	Male = 7	Professional bachelor’s degree = 78.9%	*n* = 4
	30–39 = 10.5%	Female = 12		
	40–49 = 51.9%			
	50–59 = 26.3%			
	60–69 = 5.3%			
Social work employee (*n* = 17). Total number of participants who started the training was 15 (*n* = 15)	20–29 = 14.3%	Male = 2	Professional bachelor’s degree = 64.3%	*n* = 2
	30–39 = 21.4%	Female = 15		
	40–49 = 50.0%			
	50–55 = 7.1%			
	60–69 = 7.1%			

Drop-out rates and reasons: Pedagogical staff (*n* = 0), Social work staff (*n* = 2). Reasons given: (*n* = 1), was also taking another course at the same time as this intervention and chose to discontinue the TIC intervention, and (*n* = 1) felt uncomfortable with the meditations and discontinued the intervention.

After the 8-week training employees, including the ones that had dropped out after the intervention had started, received an e-mail about the possibility of participating in an interview where they could share their experiences of the training. The email stated that if employees wanted to participate in an interview they could reply to the email. The interview would be conducted at their workplace or online, using the online platform Zoom, within normal working hours. In total four pedagogical staff members (*n* = 4) and two social workers (*n* = 2) replied to the email and agreed to participate. Upon agreement they were contacted by two of the authors (AOB, ELH), who provided them with oral and written information regarding the interviews, the data collection process, confidentiality, and data storage. Participants were given time to read the information and decide whether they still wanted to participate in the study. They were told that they could withdraw their consent at any time. It was difficult to recruit participants for the interviews in the social work group. While reasons for this is unknown, we hypothesize that either lack of time or interest could be potential reasons. Due to the difficulties of recruiting social workers, one employee in a leadership position, who had undergone the training and who had expressed interest in participating in the interview was included, making the total number of participants in the social work group three (*n* = 3).

The adequacy of the sample size was assessed *post hoc*, after data collection. Information power was applied retrospectively to determine whether the sample provided sufficient depth and relevance in relation to the study aim. Such a post hoc assessment has implications by limiting the opportunity to adjust the sample during data collection to strengthen information power, and it therefore required a reflexive evaluation of data adequacy after the analysis. Moreover, a retrospective assessment of information power carries a potential risk of confirmation bias, as adequacy is judged in light of the completed analysis rather than during the ongoing data collection process (Malterud et al., 2016). Despite these potential risks, the sample size was judged to be adequate. The demographic variables included 2 males, 5 females, and age ranging from 25 to 53 years. The participants were similar in terms of education, with all participants having a degree in either pedagogical education or social work studies. The participants within the pedagogical staff group varied in years of work experience from 5 to 10 years, while the social workers’ work experience varied from 0.5 to 14 years.

### Interview procedure

The study was registered at Aarhus University no. 2022–0367531, 3,193. Seven interviews were conducted. Five of them were conducted in person at the participants’ workplace, while two were conducted online via Zoom. Participation in the study was based on informed consent and participants were anonymized. The study’s topic was not inherently sensitive. However, because the interviews touched on stress in the workplace, AOB and ELH were aware that some participants might feel uncomfortable speaking critically about their work environment. To reduce this risk and to ensure confidentiality, all interviews were conducted individually in closed, private rooms at the workplace, ensuring that neither colleagues nor managers were present or able to overhear the conversations. The same held true for the two online interviews. The interview guide focused primarily on participants’ engagement with the intervention, their reflections on key concepts, and their use of the meditations and tools taught in the intervention after participation. Discussions of psychosocial working conditions were only briefly addressed in the interview guide, leaving participants to raise such topics at their own initiative. When stress was addressed, the emphasis was on their subjective experience rather than organizational critique. These measures were intended to support a sense of psychological safety and minimize perceived pressure related to hierarchical or collegial dynamics, while also sharpening the analytic relevance of the data in terms of the study objectives. Nonetheless, we acknowledge that the workplace setting may still have influenced how candidly some participants chose to speak, and this is considered a potential limitation in relation to data depth and openness. The semi-structured interviews were conducted with the support of an interview guide, developed in advance of the interviews. The design of the interview guide was based on the two objectives of the present study, as well as reviewed literature on factors influencing compassion training (e.g., Sinclair et al., 2021).

The two first authors (AOB and ELH) conducted this study as part of a master’s thesis in collaboration with the Danish Center for Mindfulness, Aarhus University, with no additional professional or institutional ties to the field beyond academic interest in compassion-based interventions. Although AOB and ELH were not involved in the design or delivery of the TIC intervention, their engagement with the broader research project and interest in the topic may have shaped their preconceptions. Therefore, they approached the interviews and subsequent analysis with reflexive awareness of how their attitudes and expectations could influence both the interaction with participants and interpretation of the material. As external interviewers with no prior relationship to the participants or their workplace, AOB and ELH aimed to create openness and minimize role-related bias by being transparent about their position and remaining attentive to how their involvement might affect the co-construction of data. To further mitigate potential influence, AOB and ELH deliberately avoided leading questions and refrained from expressing personal judgments. For instance, when participants discussed their experiences with the TIC intervention, AOB and ELH encouraged elaboration in the participants’ own words rather than offering interpretations. Nevertheless, AOB and ELH acknowledge that their presence and engagement may have influenced the discussions. Participants may have been subtly affected not only by the interviewers’ interest, and reactions, but also by their connection to the Danish Center for Mindfulness at Aarhus University, which delivered the TIC intervention. An effort to minimize last author (NHH) potential influence on data collection and interpretation was also considered, as NHH has extensive knowledge of practicing compassion and has conducted research on compassion-based interventions prior to this study. Therefore, NHH was not involved in developing the interview guide, in conducting the interviews, or analyzing the results. Yet as participants had met NHH, when she came to the workplace and provided information about the study, it may have affected them inadvertently in their willingness to share or not share their perspectives and subjective experiences. The interview guide can be found in Supplemental material.

The interviews were recorded with written consent from the participants. The audio files were transcribed, anonymized, and securely stored in Aarhus University’s secure one-drive system in accordance with the Danish data protection agency and Aarhus University’s GDPR guidelines, University A (2024). Participants were informed that if they experienced any adverse effects from the interview process, they could speak with a psychologist (NHH). The transcription process was assisted by Whisper AI (OpenAI, 2023) which is an automatic speech recognition system. This tool provided rough transcript drafts, which were later refined manually by two of the authors (AOB and ELH). As thematic analysis requires rigorous “orthographic” transcription (Braun and Clarke, 2006), the interviews were thus transcribed verbatim, with the inclusion of non-verbal utterances such as “uhm”, pauses, and laughs.

### Data analysis

Thematic analysis was adopted as the methodological framework to elucidate and systematically organize the participants’ subjective experiences. This approach entails a rigorous process of identifying and interpreting patterns that recur within and across the dataset. While thematic analysis served as the concrete analytic method, the study was grounded in a phenomenological epistemological orientation, since TA in itself is considered a-theoretical. This means that during coding and theme development, our focus was on participants’ subjective experiences, thoughts, and feelings about the intervention and its theoretical and practical concepts, with the phenomenological stance guiding our interpretation and prioritization of lived experience (Willig, 2013).

Thematic Analysis involves searching for and identifying patterns within and across an entire data set (Braun and Clarke, 2006). The analysis process was guided by Braun and Clarke’s (2006) guidelines for thematic analysis and conducted by two of the authors (AOB and ELH). The six steps involved: 1. Familiarizing with data; 2. Generating initial codes; 3. Searching for themes; 4. Reviewing themes; 5. Defining and naming themes; and 6. Producing the report. The authors divided the interviews between them based on groups. One transcribing, generating codes, and searching for themes within the pedagogical staff group, while the other did the same for the social worker group. After this, the authors came together to generate a thematic map across the groups, checking if the themes worked in relation to each coded interview, as well as for the entire dataset, in accordance with Braun and Clarke (2006). The themes were thereby refined, defined, and named, before extracts from the interviews were chosen and the result section of the present study produced.

## Results

**Figure 2:**
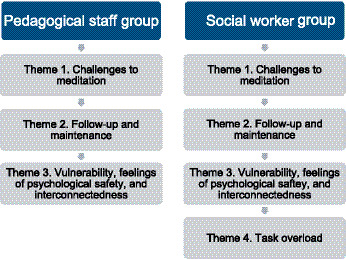
Four themes related to the implementation of TIC within the workplace and concern of the acceptability of TIC.

The data were analyzed to explore experiences with compassion training in the workplace, as well as the perceived inhibiting and facilitating factors in the implementation of TIC. Additionally, the analysis examined professionals’ perceptions of (self-) compassion and how they applied it in their daily lives, both at work and privately. The thematic analysis generated seven major themes, that were consistent across the seven participants. Four of these were grouped as relating to the implementation of TIC within the workplace and concern of the acceptability of TIC among the participants as shown in Figure 2.

**Figure 3:**
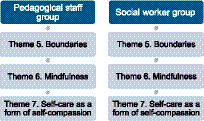
Three themes were related to the understanding and use of (self-)compassion.

The following three themes were related to the understanding and use of (self-) compassion and are presented in Figure 3.

Regarding the general acceptability of TIC within a workplace setting, five of the seven participants shared that they had an overall positive experience with the intervention, while two participants described their experiences with TIC in more negative terms and found the intervention, as a whole, to be challenging. The following themes presented here are considered potential challenges to the implementation of TIC within the workplace. Practical anonymity measures have been applied during quoting, omitting names, ages, and which group the participants belong to. The only times group membership has been kept is if there is a difference in experience between the two groups, allowing for cross-group comparisons.

## Theme 1: challenges related to meditation

The thematic analysis revealed that the meditation practices were central for the professional’s experience of TIC. More specifically, six out of seven participants expressed that meditation was challenging in some way. Two of which expressed that these challenges made participation difficult, in terms of following the program. In general, all participants thematized the issue of how to practically implement meditation as a daily habit, and how this was challenging because it required a degree of discipline and a willingness to prioritize it.

Beyond this, challenges related to meditation differed across participants. Some experienced physical or mental discomfort during meditation. For example, one participant felt nauseous while meditating. Furthermore, it was perceived as challenging to find outer peace (i.e., a quiet place to meditate) and inner calm to do the meditations. This was related to an experience of high demands from the environment, making it difficult to find alone-time in a hectic everyday life, or it could be challenging to find calm in one’s own head, reflected by experiences of racing thoughts during the meditations. One participant explained:

“My thoughts were constantly... all over the place. I couldn’t... sit and concentrate. I was sitting there, and ‘okay, then I have to do something afterward, then I have to do that, then I have to...’ You know... in my own thoughts. So I couldn’t be present.”

One of the participants described a feeling of “losing control” while meditating, and that the participant often chose not to meditate, because he/she was afraid of losing track of his/her thoughts and mental plans if he/she “gave him/herself a break,” especially in context of work. The participants had little to no experience with meditation before TIC, so the experience of meditation itself felt unfamiliar and foreign:

“My own culture, my own upbringing. So my entire, my entire childhood and my entire way... Of being raised and so on... You don't do these things. I mean, you, you, you don’t listen like that, to your body, and what... It's always moving forward, it’s, I mean, like that. It... It’s very distant in... the way I’ve been, been raised and, and grown up, right?”

This implies that upbringing, culture, and societal norms could play a potential role, in terms of why some people find meditation difficult. Different challenges with meditation made participation and commitment to the program and the training difficult and could therefore represent an inhibiting factor for the implementation of TIC.

## Theme 2: follow-up and maintenance

The second theme from the analysis is related to initiatives regarding follow-up and maintenance of the competencies learned from the TIC intervention. Six out of seven participants called for some sort of follow-up on the intervention. There were different views on whether this should be a personal or institutional responsibility. Some expressed a desire for the workplace to take the initiative to ensure that the skills from the intervention was consistently practiced and the knowledge implemented. One participant continued to practice compassion meditations daily after the intervention ended but still wished for systematic and collective follow-up by the workplace. Two participants wished for a greater leadership initiative to maintain a heightened focus on compassion as an antidote to stress after the intervention:

“At the end, we had a discussion about how we could ensure the implementation of the meditations after the course had ended. We had many good ideas about it. But they just weren’t put into practice. And then I don’t know if, if one were to do this at a workplace again, whether the management should take over, or if something should be done to ensure that it is somehow carried forward. But at the same time, you can’t force people to do it.”

The intention of the workplace to devote time and money to something that was not followed up on was questioned among several participants. Some also expressed a concern that the professional and personal benefits would disappear without follow-up and maintenance.

“But things disappear, and knowledge fades. (...) one must practice things to maintain them.”

Most of the participants found the intervention too short, arguing that the training and competencies taught in TIC are challenging and need more time than 8 weeks to master. For example, one participant highlighted the practice of giving compassion to someone one dislikes as an especially demanding task. This was linked directly to work, where it is not uncommon to handle cases where people for different reasons may have strong emotional reactions and show hostility. Altogether, this theme suggests that a lack of follow-up and maintenance at the workplace, as well as individually, may be an inhibiting factor for the successful implementation of TIC. Especially considering the potential for long lasting positive effects of the intervention.

## Theme 3: vulnerability, feelings of psychological safety, and interconnectedness

The third theme related to the implementation of TIC, concerned the feelings of psychological safety and interconnectedness in the group, and the participants’ experiences of being vulnerable with coworkers, leadership, and the TIC instructor. Five of the seven participants experienced it as unproblematic to be open and vulnerable within the group. Two participants experienced an initial resistance, because it felt strange and unfamiliar to be vulnerable with coworkers and leadership. Still, they overcame this obstacle early in the intervention, which they described were a consequence of feeling safe within the group. These participants expressed high satisfaction with the TIC instructor and felt that the instructor had a significant role in creating a safe space. The interventions’ positive consequences for the social well-being at the workplace and feelings of interconnectedness within the group was described like this by one participant:

“So, uh, when we had to talk about how the week had been, and stuff... It was insanely honest. And that, I mean... I don’t know, I wasn’t prepared for that. That, that people would be so honest and say... so many, nice things about how they had experienced all sorts of things, right? Uh... So it was really a... it was such a huge eye- opener in some way. I think, for the whole group.”

One participant from each group found the intervention to be especially challenging in general and did not feel completely acknowledged by their TIC instructor, in terms of their difficulties with the content of the program. While one participant had wished for closer, individual follow-up from the instructor, another participant reported that the instructor could have placed greater focus on challenges of meditation and normalized this participant’s experience to a higher degree. The participant stated that it had felt somewhat lonely to sit with the experience by him/herself. These perceptions were related to the participants’ challenges to be open with the rest of the group:

“I needed something more, which wasn’t there. I mean, I had... uh... And I also said to... to the instructor, that I kind of... I had wished, it would have been a big help for me... If every time we had this once a week, I could spend 5-10 minutes with the instructor, alone. I mean, to be pulled out of this common circle. And asked directly with... ‘so how are you doing, how are the exercises going, can I help you with something personal...’ Because, it’s a bit boundary pushing to... to express how one feels in such a large uh... Circle, right?”

It was also addressed that the inclusion of the leadership in the intervention groups, contributed as an additional challenge to be open and feel safe to share with the group:

“Because I really wanted to feel that it was super cool and... for my leaders to see that I was really good at mindfulness and compassion, but that wasn’t my experience. I thought it was really tough, and... I thought it was really hard to be in. And I found that a bit difficult to say, while the leaders were sitting there.”

This theme suggests that for learning to happen, psychological safety within the group is paramount. Based on these participants’ experiences, challenges to creating psychological safety may be related to power hierarchies within the group and perceived warmth of the instructor. These variables may challenge a positive experience of interconnectedness within the group and perceptions of personal gain from the intervention.

## Theme 4: task overload

While the two participant groups’ work tasks and work structure differed, the three themes related to challenges of implementing TIC within the workplace, were consistent across the two groups. However, an additional implementational challenge was present in the social work group. This challenge related to lower participation, completion, and higher drop-out rate during the TIC intervention. As revealed by the interviews, the stress this professional group experience in their workday is related to a large workload, deadlines, and the emotional burden of feeling responsible for peoples’ lives. This includes handling the possible negative reactions they meet because of their professional decisions. The social work group hoped that TIC could provide helpful tools for their team in terms of handling stress. Unfortunately, two of the social workers, who had signed up to participate in the intervention were on sick leave due to stress in the period the team received the intervention. Others felt that they did not have the time to participate in the weekly program and therefore only showed up to some of the session. Consequently, only half of the team participated in TIC, two of who dropped out early in the intervention. The three social workers, who chose to participate in the interviews, all completed the TIC intervention. However, the low participation in their team still impacted their experience of the intervention. They expressed frustration on behalf of the team, that not more could benefit from the training. They also felt that the training did not lead to the expected changes in the psychosocial work environment, as their perception was that those who might have benefited the most from the training were also the ones who did not participate. One participant described the issue like this:

“And then it became more of a stress factor to participate, than to be... So, in that way, it had a somewhat opposite effect. And I also think, some of them chose to say that they couldn’t join, because it didn’t bring them any calm but rather made them feel stressed about prioritizing the time. There were many hours to prioritize. But we did have support from our area manager to do it. But it doesn’t always change the feeling you have inside (...).”

This illustrates a paradoxical problem of offering interventions at workplaces to prevent and mitigate stress, as prioritizing the time and commitment to an eight-week program during the workday may be perceived as a stress factor itself.

### Understanding and use of (self)-compassion

Our analysis indicated that the concept of ‘compassion’ was an unfamiliar construct for the participants in advance of the intervention. Across the groups, the participants understood compassion as being kind and respectful to others as well as to oneself. Furthermore, they understood compassion as being able to handle difficult situations, while still being empathetic and caring. The following three themes concern the participants’ understanding and use of (self-) compassion in their daily lives.

## Theme 5: boundaries

The analysis revealed that drawing boundaries was a central theme related to the participants’ understanding and use of compassion. This was expressed in different ways. Among the pedagogical staff, boundaries were discussed in relation to the issue of being able to maintain a healthy work-life balance:

“Therefore, I have learned at least that, to take care of myself, and... when I take a vacation, I need to get away. And then turn off... the phone.”

In addition to setting boundaries in terms of work-life balance, participants in the social worker group also discussed boundaries in relation to an extensive workload, allowing oneself to be satisfied with the work one has done, even in the face of strong emotional demands from others:

“So, I try not to judge whether... yes, whether their feelings are right or wrong. But those are the feelings they have, and that's how it is. And so we can’t... that thing about, now I have to tell myself ‘I can only do so much here’, and ‘I can’t do more than that’.”

Participants across the groups felt that being able to set boundaries when engaging with clients’ emotional reactions was essential for showing compassion both towards others and themselves. They considered it an important skill to be able to act, when they experienced empathetic distress towards someone else’s emotional reactions, rather than being paralyzed by strong emotions. One participant explained this as a “filter” being added, which implied an ability to separate oneself from the other. Another participant described this ability as a form of “professional love.” The distance implied in professional love was described as “distance without distance,” beneficial both for the service user and for the professional him/herself, in terms of being able to have the clarity and strength to help the person, and at the same time avoid empathic distress. This self-other distinction was described by one participant as “being more inside oneself, and less inside the other.” A participant described compassion as a way to “avoid empathic distress.” Further, another participant described it like this:

“Indeed, not to internalize others’ feelings. Rather, to be there and help them, without taking their emotions with you. Because I believe... that’s really important, not doing so in this work. Otherwise, I think you’ll become compassion fatigued.”

## Theme 6: mindfulness

The practice of mindfulness and the experiences of being mindful was a recurring theme regarding how the participants understood and used compassion in their daily lives. The participants experienced positive outcomes from the meditations in different ways. Mindfulness was experienced as a tool that allowed them flexibility, energy, and adjustment between tasks. For example, one participant described it as a “shift between worlds.” Another described it as a way of giving oneself a “mental hug,” which helped the participant feel ready to face difficult situations at work. For others mindfulness was about listening to themselves and being able to notice and connect with their bodies, as well as getting out of their heads:

“Well, I think, that state of flow one enters when practicing mindfulness, where you feel a lot in your body, and maybe also detach a bit from the mind, feeling what’s happening in your body. I find that I have been able to use that.”

The analysis revealed experiences of increased calm and inner peace as a consequence of practicing mindfulness:

“I think... Like... I really feel that it gave me so much. Like... personally. And a whole new calmness... inside myself. That... makes it... I come to work in a whole new way. I prepare differently. I actually do things... completely different than I did before.”

As well as an increased awareness and presence:

“Also, those everyday meditations we’ve been taught, the idea of being present in the moment. Closing your eyes and feeling the air and the sounds. (...) I think it’s a really nice thought, being present in what you’re doing.”

## Theme 7: self-care as a form of self-compassion

In relation to the participants’ understanding and use of self-compassion, the data revealed that self-love and self-care were a recurring and central theme across all participants. They discussed the experience of changing the way they talk to and relate to themselves, to becoming more kind and friendly towards oneself:

“The whole way of being self-loving towards myself, (...), also involves how I talk to myself. And how I, sort of, uh, stop beating myself up with, ‘Oh, you couldn't figure that out either, you couldn’t figure that out either’.”

One participant noticed how this change is not necessarily easy, as this participant’s “brain does not function like that.” This reflects an overall observation shared by four of the participants that having self-compassion was unfamiliar and boundary-pushing and something that had to be learned. The remaining three participants did not express these sorts of barriers towards the concept of self-compassion. The analysis indicates that TIC overall contributed with an increased awareness of self-compassion and its importance, although participants enforced it in their lives to varying degrees. Other experiences of self-compassion involved becoming more forgiving towards oneself and giving oneself comfort when needed. Concrete examples included doing the meditations practices from TIC, saying nightly prayers, and writing down comforting words in distressing situations. For many of the participants, self-compassion was about prioritizing time to oneself and taking care of oneself. One participant described it like this:

“I have decided to try compassion to see if it can give me something. So, I need to prioritize it. For me, it is very much about prioritizing **[…]**, some me- time, some alone-time, where I try something with the goal of becoming calmer.”

## Discussion

Workplaces employing healthcare workers (i.e., nurses, physicians, psychologists, social workers, educators, and other pedagogical staff), are searching for ways to address the mental health and well-being of their employees. Stress, burnout, medical leaves, and high turnover rates are not only costly but also detrimental to patients, clients and service users who are dependent on the institutions and the formal care workers employed there. This study therefore aimed to understand whether a compassion-based intervention would be helpful in addressing these concerns. Specifically, the present study aimed to investigate the experiences of training compassion and perceived inhibiting and facilitating factors in the implementation of TIC within a municipal institution in Denmark, and how participants understood and integrated the practice of (self-) compassion. The data collected from seven interviews conducted with two employee groups, are context-bound to a Danish municipal social service system and shows that the answers are complex. Participants narratives illustrate their subjective experience of the perceived impact of the intervention both on a personal and organizational level and should not be misinterpreted as demonstrating intervention effect.

The first complex issue related to variations in participants’ subjective experiences of practicing compassion. Some participants reported the practices to be life changing, understanding (self-) compassion as a skill set to become more present, to set healthier boundaries, carving out some ‘me time’ and to take better care of themselves, thereby reducing empathetic distress when engaging with service users. Their subjective understanding was that compassion was a kind of ‘professional love’ that allowed for the self-other distinction to emerge when they engaged with the service user, creating less emotional exhaustion.

In addition, some participants stated that the practices led them to feel more connected with their colleagues and feeling safe in a group setting. These results align with the mental health benefits of previous research (Hansen et al., 2021; Watts et al., 2021; Lamothe et al., 2018; Sinclair et al., 2021). Specifically, a recent meta-analysis on the effects of practicing loving-kindness in the workplace showed that employees benefitted from the practice regarding health outcomes such as; burnout, mindfulness, stress, self-compassion, job attitudes, personal mental health, psychological resources, and interpersonal relationships (Wang et al., 2024).

Other participants found the training challenging. For some, compassion felt unfamiliar. It pushed their boundaries and went against their habitual way of thinking and talking to themselves. Some found it difficult to connect with and understand the word compassion, and some shared that they experienced loneliness, as they were having difficulties with the meditation practice and did not feel seen by the TIC instructor. Some found it difficult to find physical, cognitive, and emotional stability when engaging with the meditative practices, and some felt unsafe in the larger group due to the unfamiliarity of sharing personal experiences with employees and/or because of the power imbalance between employee and leadership. These results also aligns with previous research (Hansen et al., 2025; Jazaieri et al., 2016).

These differences in participants experience of the practices are not unique to the participants in this study, but is shared time and again, whether the practices are introduced in a workshop at a workplace or in a course open to the public (Hansen et al., 2025). In general, people’s experiences with compassion and the meditations varies, peoples sense of feeling safe in a group varies, and peoples understanding of concepts varies (Sinclair et al., 2022). These shared individual differences may point to a larger issue of whether compassion training, practiced in a group setting through compassion meditation practices, is the ‘best’ way to introduce compassion into a workplace setting. Not only does it take time to implement a new practice into everyday life, but compassion meditations are also inviting people to engage with suffering (i.e., a difficult relationship, difficult emotions, thoughts, or physical sensations). The question remains whether meditation is the ‘best’ way to practice compassion in a workplace setting, and whether inviting colleagues to share potentially personal and sensitive information with each other in a workplace setting is ethical?

Regarding the participants’ understanding of the construct of compassion, a pronounced emphasis on self-care and personal boundaries was seen. This emphasis was not an explicit aim of the intervention design but rather reflects the aspects that participants themselves highlighted as most meaningful to them. As a result, the relational dimension of compassion—particularly the capacity to recognize and respond to the suffering of others—receives less attention in the analysis. This may indicate that the study foregrounds the self-directed component of the compassion construct, to a greater extent than its interpersonal dimension. A disproportionate focus on the self-compassion component of compassion is a known critique of secular compassion interventions, which potentially neglects the interconnected experiences of compassion (Quaglia et al., 2021). At the same time, the fact that participants spontaneously emphasized self-care and boundary-setting may also suggest that these elements are experienced as a necessary foundation for sustaining compassion towards others in demanding caregiving roles. In this sense, the findings could be interpreted as pointing to an important prerequisite for a sustainable compassion practice.

The theme of maintenance and follow-up was raised by participants, not only in terms of wanting to continue to practice but also as an uncertainty regarding who is responsible for the continued practice and use of the newly acquired skills. Research shows that for people to maintain the positive effects of the practice on their mental health, continued practice is needed (Sinclair et al., 2021), yet how to go about doing this or implementing this in a workplace setting remains unknown. Perhaps, one way to address the issue would be to hire and/or educate an employee who has the required education and skills to continue to guide employees, when they encounter difficulties in their practice or how to use their practice in their daily lives. Research suggest that having a skilled compassion instructor is key to implementation (Sinclair et al., 2022). Yet, cross-group comparisons showed that the social work group felt that the intervention was an additional stressor to an already stressful workday, and may therefore, not have wanted to continue to practice even if the organization had offered continued practice. Therefore, understanding what kind of employee group will benefit from a mental health intervention in the workplace and who will not, is an important question to ask before implementing an intervention.

The theme of psychological safety, interconnectedness, and vulnerability within the group, was a key challenge in implementing a compassion-based intervention in the workplace, especially given the mix of vulnerability and power dynamics within the group. Including leaders can be highly beneficial, as they may model vulnerability, common humanity, and interconnectedness, thereby strengthening psychological safety and supporting employee well-being (Farr and Barker, 2017). However, their presence may also heighten feelings of exposure or insecurity for some participants and create a barrier to engagement. Research shows that leadership support is crucial for successful implementation (Paakkanen et al., 2024; Sinclair et al., 2021; Curtis et al., 2017), but it remains unclear whether leaders should participate in the training alongside employees. To address the ethical concerns, organizations could establish clear consent guidelines or offer separate sessions for leaders. Individual experiences to the practices also varied: some participants found the practices uncomfortable, while others described them as life changing. This suggests that while not everyone will benefit equally from workplace mental health interventions, the gains for some justify continued implementation. Ongoing training and support may help participants work through challenges and sustain the benefits of compassion-based practices (Paakkanen et al., 2024; Sinclair et al., 2021; Curtis et al., 2017).

A third complex challenge that was experienced by the social worker group but not the pedagogical staff group was the theme of task overload. Participants in this group, did not feel that they had time to engage in the intervention, and many chose to skip the sessions to have “enough” time for other work tasks. The idea that those who needed the training the most were also the ones who did not show up, highlights a larger issue at hand. When implementing an intervention that is aimed at reducing poor employee mental health, the dilemma of whether to conduct the intervention within normal working hours or outside of normal working hours arise, usually as a question or concern with cost. If employees are allowed to participate within normal working hours, what happens to the work that is not being done while they are participating in the intervention? If they are invited to participate in the intervention outside normal working hours, will they sign up or choose “free time” instead?

The theme of task overload, which was specific to the social worker group, was stated as a reason for not attending the intervention every week. While everyone who signed up for the intervention in the pedagogical staff group participated and completed the training (19 participants completed at least six of the eight session), only half (7 out of 15) of the social work participants showed up to training each week and half participated in less than five sessions (8 out of 15). Of the eight, one dropped out because he/she was participating in another course at the same time as the intervention, and one dropped out due to feeling uncomfortable with the meditations. While task overload may be one reason for the differences in participation, age and work experience may be another. In the social worker interview group, some were in the age group 20–29, which was not seen in the pedagogical staff interview group. Some were new to the job in the social worker interview group, which was also not the case in the pedagogical staff interview group. Moreover, the two groups had different leaders and different TIC instructors, and as mentioned their roles and responsibilities when engaging with the service users differed. These cross-group differences may have played a role in their experience of having or not having “time” to participate. Recent research on loving-kindness training in the workplace shows that its effects can differ depending on a person’s gender, job role, and the specific focus of the practice (Wang et al., 2024). Other research point to age playing a role, such that older employees experience less burnout, emotional exhaustion, depersonalization, and a greater sense of personal accomplishment, compared to younger employees (Hybels et al., 2022).

In considering these cross-group comparisons, it would be interesting to understand the issue of “task overload” and empathetic distress. One could argue that task overload also holds true for the pedagogical staff, yet this did not show up in the interviews for this group. The pedagogical staff operate as the glue between the municipality, the service user, his/her family, and the school. It may be that the idea of task overload and the empathetic distress experienced by pedagogical staff is more related to never feeling like they are “off from work” as suggested by one participant, whereas task overload as it is experienced by social workers is related to the feeling of not having enough time to do their work properly. This could further be related to the theme of self-care as a form of self-compassion, where cross-group comparison showed that the “setting of boundaries” were applied or understood differently. For the pedagogical staff group setting boundaries were related to bringing a case/service users problems home or leaving work at work. For the social worker group setting boundaries were related to managing the task load or having an understanding that one had done “enough” for a particular service user.

Finally, while individual differences for engaging with, or finding it difficult to engage with the (self-) compassion practices emerged in the participants subjective experiences, participants in the pedagogical staff group agreed that the main barriers to providing compassionate care were primarily due to systemic constraints and bureaucratic obstacles, rather than the emotional demands of interacting with clients. This is in line with the results of a recent study investigating the barriers to compassion, which found that one of the barriers to bringing compassion to the fore in organizations, is the notion of efficiency being more important than well-being. This was expressed as stress, excessive workload, insufficient or inappropriate use of resources, and bureaucratic inflexibility (Paakkanen et al., 2024). Among the social workers, task overload, as mentioned above, was discussed as a main barrier to well-being at work and resources to provide compassionate care, in addition to handling clients’ distress. This resonates with Tanner (2020), who argues that the emotional risks faced by social work professionals are not primarily due to emotional engagement with clients’ distress but are rather due to contextual constraints that inhibit their motivation to alleviate it, underscoring the role of organizations in facilitating this motivational aspect of compassionate care. Individual differences, psychological safety, self-care, task overload, leadership, and institutional structures seem to all play a role when addressing employee health and well-being and should be addressed when thinking about who may or may not benefit from compassion -based training in the workplace.

### Limitations

This study is not without limitations, and several factors may have influenced the results. First, this data is built on a very small sample size. We were only able to recruit seven employees who were willing to participate in the interviews. As stated, information power was evaluated post-hoc, making it difficult to ascertain whether different experiences would have emerged had more interviews been conducted or whether saturation was achieved. Moreover, the *post hoc* assessment had implications by limiting the opportunity to adjust the sample during data collection. Had more employees been willing to participate in the interviews, it would have allowed for sample adjustment and thereby strengthening information power. In addition, a retrospective assessment of information power carries a potential risk of confirmation bias, as adequacy of the data, is judged in light of the completed analysis rather than during the ongoing data collection process (Malterud et al., 2016). A small sample size also allows for the risk of having overinterpreted the data, giving too much weight to individual experiences and reflections, or that single participants have disproportionately shaped the themes.

Secondly, information bias may have been present as the employees volunteering to participate could be ones, who were heavily invested in the training and/or in the workplace. This sample may therefore not be a representative sample of the employees and their experiences with the training. Therefore, the themes may not be generalizable to the entire workplace or transferable to other contexts, settings, and groups.

Third, the context of the data collection (qualitative interviews) represents a social interaction where factors such as social desirability might influence the responses of participants and their potential willingness to share critical perspectives (Bergen and Labonté, 2020). Therefore, social desirability bias may be present.

Forth, as five of the interviews were situated at the professionals’ workplace, it may have played a role in how safe they felt to share their honest opinions regarding their experiences. Moreover, two of the interviews were conducted online via Zoom, which may have influenced participants’ sense of psychological safety, either positively or negatively. Previous research supports this observation, suggesting that the online format can enhance participants’ sense of control and safety (Oliffe et al., 2021).

Fifth, the gender distribution among participants was skewed towards women, potentially providing a less balanced perspective on the training than if more men had participated. This reflects a common trend in studies on MBIs and CBIs, which frequently report a higher proportion of female participants (Kirby et al., 2017; Lamothe et al., 2018; Sinclair et al., 2021). This highlights the importance of deliberately exploring men’s experiences with TIC, and similar interventions, in future research.

A sixth limitation of this study relates to sampling bias. As this was a convenience sample, it unfortunately did not include participants who did not complete all eight sessions or discontinued the intervention. While these participants were similar regarding, gender, age, and ethnicity to the ones who did participate, we do not know how they experienced the intervention. Therefore, we do not know if the same or different themes had emerged had they participated. A final limitation to the study includes recall bias, as participants are self-reporting on the changes they experienced with the practices, which is common in retrospective qualitative designs.

## Conclusions and future directions

Overall, the findings of this study highlight the complexities that must be addressed when deciding to implement an intervention that aim to increase employee well-being and decrease employee poor mental health resulting in stress, burnout, sick leave, and high turnover rates. Complex issues such as whether compassion can be implemented and practiced in other ways than through meditation, ensuring psychological safety of the employees, how to implement maintenance and follow-up training, and issues with task overload must be addressed to circumvent some of the challenges to the implementation of compassion training in workplace settings.

Future research should examine long-term outcomes and sustainability of MBIs and CBIs, which will be crucial for understanding their full impact on formal care providers’ well-being and professional effectiveness and could potentially be done using prospective designs to understand participants experiences, and the processes as they unfold. Moreover, using an organizational change model such as the Consolidated Framework for Implementation Research (CFIR) could help to analyze how *compassion training* is implemented in an organization by identifying barriers and facilitators at multiple levels (e.g., leadership support, workload, staff attitudes) (Damschroder et al., 2009). Addressing the challenges identified and leveraging the facilitators will be essential for optimizing compassion training interventions and ensuring they meet the needs of formal care providers. By doing so, these interventions can contribute significantly to the well-being and resilience of professionals in high-stress environments, ultimately improving the quality of care they provide.

## Data Availability

The qualitative datasets generated and analyzed during the current study are not publicly available due to concerns about participant confidentiality, but de-identified excerpts are available from the corresponding author on reasonable request. The interview guide used in the present study, is available in Supplemental material. Requests to access the datasets should be directed to elinehakedal@hotmail.com.

## Footnotes

1 (Self-)compassion refers to the duality of the compassion training. In the formal meditations participants practice giving compassion to themselves, loved ones, neutral people, difficult people and all sentient beings.
