# Supplementary material for: A qualitative investigation of social service workers’ experiences with compassion training in the workplace
Source: Front Psychol. 2026 Feb 2;16:1593026. doi: 10.3389/fpsyg.2025.1593026 (PMC12910161; doi:10.3389/fpsyg.2025.1593026)
Supplement: Supplementary file 1 [file Table_1.DOCX]

Supplemental Material 1

**Interview Guide**

**Introduction**

- Brief presentation of ourselves and the project
- Duration: up to one hour
- Information about consent, anonymity, and audio recording

**Background Questions**

- How old are you?
- What kind of education do you have?
- How long have you been employed at your current workplace?
- Please describe your usual work tasks during a typical day.
- What do you particularly enjoy about your job?
- What motivates you to work in your current field?
- Is there anything you enjoy less about your job?
- Have you experienced stress or dissatisfaction at work?
- To what extent does this affect your workweek?

**Main Questions**

**General experience of TIC**

- What thoughts or expectations did you have before starting TIC?
- Have you noticed any changes in the psychosocial work environment after TIC?
- Have you noticed any changes in your well-being?
- Have you noticed any changes in your stress level?

**Understanding of compassion and transfer of TIC to real life**

- How do you understand the concept of compassion?
- What does self-compassion mean to you?
- Has your understanding of compassion changed after participating in TIC?
  How and in what way?
- How did the instructor/therapist contribute to your understanding of compassion?
- Do you use what you have learned from TIC?
- Do you experience using compassion for others in your professional context after TIC?
  If yes, in what way? What does it look like in practice?
  If no, what do you think is the reason for that?
- Do you experience using self-compassion in your professional context?
  If yes, in what way? What does it look like in practice?
  If no, what do you think is the reason for that?
- Do you generally give yourself compassion during the week?
  If yes – why / what is your motivation? What does it look like when you practice compassion?
  If no – why not? What do you think prevents you?

**Questions about the experience of the course implementation**

- How was it to practice compassion?
- What was the most challenging part?
- What was the most educational or useful part?
- How was it to practice compassion together with colleagues?
- How was it to practice compassion when managers were present?
- What was most important to you during the course period?
- How did your workplace or employer facilitate your participation in the course?
- Is there anything you think could have been done differently?
